# Internal Jugular Vein Thrombosis Following Different Types of Neck Dissection

**DOI:** 10.22038/ijorl.2020.25549.1839

**Published:** 2020-11

**Authors:** Maziar Motiee-Langroudi, Amin Amali, Babak Saedi, Iraj Harirchi, Sedigheh Hasani, Leyla Sahebi, Mahtab Rabbani Anari

**Affiliations:** 1 *Otorhinolaryngology Research Center, Tehran University of Medical Sciences, Tehran, Iran.*; 2 *Department of Oncologic Surgery, Cancer Institute, Imam Khomeini Medical Complex, Tehran University of Medical Sciences, Tehran, Iran.*; 3 *Maternal-Fetal and Neonatal Research Center, Tehran, University of Medical Sciences, Tehran, Iran.*

**Keywords:** Jugular vein, Neck dissection, Thrombosis

## Abstract

**Introduction:**

During functional neck dissection, the surgeon tries to preserve the internal jugular vein (IJV); however, the incidence of its narrowing or obstruction following modified radical neck dissection (MRND) or selective neck dissection (SND) varies between 0% and 29.6%. The most distressing complication of IJV thrombosis (IJVT) is pulmonary embolism. This study aimed to evaluate the incidence of IJVT following selective or modified radical neck dissection.

**Materials and Methods:**

In this study, 109 neck dissections were performed with the preservation of the IJV on 89 patients from March 2011 to December 2012 in the Cancer Institute of Imam Khomeini Hospital Complex, Tehran, Iran. Ultrasound evaluation of the IJV was performed in the early postoperative period and three months after the surgery.

**Results:**

The study population consisted of 62 male and 27 female patients with a mean age of 57+17.57 years. Ultrasound evaluation of the IJV among the participants (109 veins) indicated thrombosis in nine veins (8.25%) in the early postoperative period, four of which remained thrombotic and without flow three months after the surgery. Moreover, 96.33% of the IJVs were patent with a normal blood flow three months after the neck dissection. Among the evaluated IJVs, the only factor that showed a significant association with IJVT was the incidence of postoperative complications, including hematoma and seroma (P=0.01).

**Conclusion:**

It seems that the most important factor for the prevention of the IJVT is a meticulous surgery and surgical complication avoidance during neck dissection.

## Introduction

Ligation of the bilateral internal jugular veins (IJV) during radical neck dissection can induce serious postoperative complications, such as facial edema, intracranial hypertension, chemosis, optic nerve complications, and the syndrome of inappropriate antidiuretic hormone secretion that can be a cause of death.

The term "modified radical neck dissection" was described in the literature by Suarez in 1963. Functional neck dissection aims to reduce the significant long-term morbidity and deformity due to sacrificing the sternocleidomastoid muscle, accessory nerve, and IJV, especially if it is bilateral (1-3).

During functional neck dissection, the surgeon tries to preserve the IJV. However, the incidence of IJV narrowing or obstruction following modified radical neck dissection (MRND) or selective neck dissection (SND) varies between 0% and 29.6% in different studies and can cause the same complications mentioned for the IJV ligation (4-6). 

The most dreadful complication of IJV thrombosis (IJVT), although rare, is pulmonary embolism (6,7). Some risk factors for the IJVT are technical difficulties during vessel dissection, such as the way of branch ligation, thermal injury to the vein induced by electro-cautery, and desiccation after fascia detachment (4). This prospective study aimed to evaluate the incidence of IJVT following selective or modified radical neck dissection. Moreover, it was attempted to investigate the effect of controversial risk factors, such as postoperative anticoagulant therapy, history of neck radiotherapy (8,9), repair of the small jugular ruptures, and surgical complications.

## Materials and Methods

**Fig 1:**
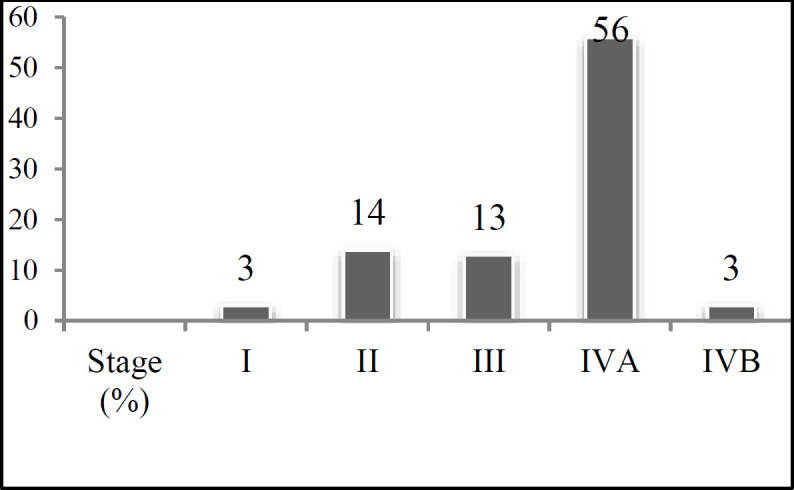
Stage of tumors before surgery among included patients in the Cancer Institute of Imam Khomeini Hospital Complex, Tehran, Iran (n=89)

In this study, 109 neck dissections were performed with the preservation of the IJV on 89 patients from March 2011 to December 2012 in the Cancer Institute of Imam Khomeini Hospital Complex, Tehran, Iran. The mean age of the patients was 57±17.57 years (age range: 18-88). The majority of the patients were male (n=62), and informed consent was obtained from all patients before participation in the study. All patients had patent jugular veins and underwent neck ultrasonography, computed tomography scan, and magnetic resonance imaging before surgery. Table 1 tabulates the primary site of the tumors. The staging was made based on the criteria of the *National Comprehensive Cancer Network*. Figure 1 shows the participants' tumor stages. Among 109 neck dissections, there were 57 (52.29%) selective neck dissections (level I, II, and III) sparing the IJV and 52 (47.70%) modified radical neck dissections with removing at least level II, III, IV, or V and preservation of the IJV. 

**Table 1:** Frequency of tumor sites among included patients in the Cancer Institute of Imam Khomeini Hospital Complex, Tehran, Iran (n=89)

**Primary Tumor Location**	**No. of patients (%)**
Tongue Larynx Lip Thyroid Salivary Glands Hard Palate Buccal region Mandible Floor of the mouth Other Total	31 (35) 16 (18) 7 (8) 5 (5.6) 5 (5.6) 5 (5.6) 5 (5.6) 4 (4.5) 2 (2.2) 9 (10) 89 (100)

All procedures were performed by the same surgeon. Postoperatively, the patency of the IJVs was evaluated using Doppler ultrasonography about 3 to 7 days (depending on the ambulatory state of the patient) after the operation. 

A radiologist documented the presence of thrombosis, diameter of the IJV and its blood flow, as well as other surgical complications, such as hematoma and abscess. The patients with IJVT were re-evaluated three months after surgery by the same radiologist using the Doppler ultrasonography. In total, 14 (16%) patients had undergone radiotherapy before the operation; moreover, 56 (63%) and 4 (4.5%) cases received heparin and enoxaparin after the surgery, respectively. The data were analyzed using the Chi-square test, and a p-value less than 0.05 was considered statistically significant.

**Table 2:** Comparison of some related variables with IJVT occurrence after surgery

Variables	Cofactors(n)	Occurrence of IJVT		P-value
		Yes (%)	No (%)	
**Gender**	Female (27)	4 (14.81)	23 (85.18)	>0.05
	Male (62)	5 (8.06)	57 (91.93)	
**Age (year)**	<40 (11)	0 (0.0)	11 (100.0)	-
	41-50 (11)	0 (0.0)	11 (100.0)	
	51-60 (33)	2 (6.06)	31 (93.93)	
	61-70 (20)	3 (0.15)	17 (0.85)	
	>70 (14)	4 (28.57)	10 (71.43)	
**Type of surgery**	SND (57)	5 (8.77)	52 (91.23)	>0.05
	MRN (52)	4 (7.69)	48 (92.31)	
**Postoperative complication**	Yes (10)	3 (30)	7 (70)	0.01
	No (79)	6 (7.59)	73 (92.4)	
**Anticoagulant therapy**	Yes (60)	6.0 (10.0)	54 (90.0)	>0.05
	No (29)	1 (3.4)	28 (96.55)	

## Results

Doppler ultrasonography of the IJV in 89 patients (109 veins) showed thrombosis in 9 veins (8.25%) in the early postoperative period, four of which remained thrombotic and without flow three months after surgery. Moreover, 96.33% of the IJVs were patent with a normal blood flow three months after the neck dissection. The incidence rates of the vein thrombosis in SND and MRND were 8.77% and 7.69%, respectively, with no significant difference between them (P=0.83). Table 2 summarizes the comparison of some variables with IJVT occurrence after surgery. 

There was no association between the age of the patients and IJVT although the number of cases with IJVT was higher (~28.6%) in patients older than 70 years old. The incidence rates of vein obstruction were 14.8% and 8% in females and males, respectively, which showed no significant difference between them in this regard.In this series, 10 patients developed postoperative complications; moreover, 7, 2, and 1 cases had hematoma, seroma, and chylous fistula, respectively. 

Out of these patients, three cases had IJVT five days after the surgery. The incidence rates of vein thrombosis were 30% and about 7.6% in the patients with and without postoperative complications, respectively. Therefore, there was a significant relationship between the presence of postoperative complications and IJVT (P=0.01).

Anticoagulant therapy caused no significant difference in the incidence of IJVT. Out of 60 patients who received anticoagulant therapy, the IJVT was observed in 6 cases, whereas one patient suffered from this complication among 29 patients who did not receive this therapy (P>0.05).

Totally, 14 patients received preoperative radiotherapy, and occlusion occurred in two of these cases. There was no significant difference between the groups with and without preoperative radiotherapy in terms of the rate of vein occlusion (P>0.05).

Furthermore, the analysis of the incidence of IJVT according to the primary site of the tumor did not show any significant difference in this regard. It was necessary to repair 25 IJVs intraoperatively by simple suturing; accordingly, the cases were divided into two groups. In group one, 1 to 3 points of the vein were repaired (21 out of 25 cases), whereas more than 3 points were repaired in group two (4 out of 25 cases). There was no significant difference between these two groups and the group with intact IJVs regarding IJVT (84 out of 109 cases) (P=0.43).

The incidence of vein occlusion was 8.88% in node-negative (N0) necks and 7.81% in node-positive (N+) necks with no significant difference between them in this regard.

## Discussion

The importance of the IJV patency is obvious after neck dissection, especially in bilateral dissections and in the presence of reconstruction with a flap. Although the presentations of unilateral IJVT are subclinical, the probability of inflammatory thrombophlebitis and a small risk of pulmonary embolism should be considered (10). Different risk factors are suggested for IJVT following neck dissection. The nonsurgical factors that have been evaluated in other studies include postoperative radiotherapy, use of a central catheter, external pressure from a pedicled myocutaneous flap or drain, infection, overt salivary fistulas, and wound breakdown in the area of the preserved vein (6,10). Technical factors that can prevent IJVT include meticulous dissection of the IJV, prevention of thermal injury from the electrocautery, prevention of the aridness of the vein surface once its adventitia is dissected, ligation of the IJV branches in the proper site. Ligatures that are too far from the vessel may cause blood stasis and clot formation, while those that are too close to the vessel can narrow the lumen (6,11).

The same surgeon carried out all the neck dissections in this study, and thermal vein injury and desiccation of the vein following adventitia dissection were prevented as much as possible to avoid the risk factors linked to surgical methods. It was tried to perform atraumatic dissection and accurate branch ligation of the IJV although 25 IJVs had to be repaired intra-operatively by simple suturing.

The results of this study showed an IJVT rate of 8.25% (9/109 veins) in the early postoperative period (5^th^ day), followed by a rate of 3.66% (4/109) three months after the surgery. Moreover, 96.33% of the IJVs were patent with a normal blood flow three months after the neck dissection. Harada et al. reported that after the surgery, the jugular blood flow returned to baseline increasingly. 

Additionally, the fibrosis and scar tissue of the neck stretch out the neck spaces and also may cause an incremental change in the diameter of IJVs within three months after the surgery (6). The incidence rate of IJVT ranges from 0% (76 veins) reported by Harada et al. to 29.6% (8/27 veins) in a study conducted by Leontsinis et al. (10).

In the present study, the number of dissected veins was higher than that in similar studies. Furthermore, some factors can explain the different incidence rates of IJVT in different studies. Postoperative radiation, presence of a pedicled myocutaneous flap, as well as other complications in the postoperative course may have contributed to IJVT (10). 

The definition of occlusion (partial or complete) is not well-defined in these studies. However, both partial and complete occlusions were included in this study, which means that the presence of thrombosis was investigated.

The cases of reconstruction with a flap were not investigated in this study; however, this study evaluated the early postoperative thrombosis and it’s follow-up. Therefore, the effect of postoperative radiotherapy was not investigated in this study. In the present study, among factors, such as the type of neck dissection, preoperative radiotherapy, and postoperative anticoagulant therapy, the only factor that showed a significant association with IJVT was the incidence of postoperative complications, including hematoma and seroma. No cases of infection or salivary fistulas were observed in this study. However, the effect of age was analyzed on IJVT, which has not been addressed in other studies so far. Although the percentage of IJVT in patients older than 70 years old was greater, the difference was not significant. There was no relationship between thrombosis of the internal jugular and the preoperative nodal status of the neck either, which was in line with the results of a study conducted by Brown et al. 

Among nine patients with IJVT, four cases were N0 and five individuals had nodal disease. In our study, preoperative radiotherapy did not affect thrombosis (10), which was consistent with the results of other studies. Ultrasound was utilized to detect IJVT in this study. Brown et al. reported no inconsistency when comparing the enhanced CT and high-resolution ultrasound or color-flow Doppler (4). 

In this study, the incidence of venous thrombosis was 8.77% in SND and 7.69% in MRND, with no significant difference between them (P=0.83). It seems that when the technical points, which were mentioned earlier, are considered in the neck dissection and the preservation of IJV, the extent of the dissection is not an independent risk factor. Interestingly, no significant difference was observed between the cases with simple suture repaired IJVs (25/109) and the group with intact IJVs (84/109) regarding the rate of IJVT (P=0.43). The role of postoperative anticoagulant therapy for the prevention of IJVT was not confirmed in this study.

## Conclusion

This study investigated some controversial risk factors of IJVT after neck dissection with a fairly large sample size. It seems that the most important factor is the meticulous surgery and surgical complication avoidance. In future studies, it is required to evaluate the role of postoperative radiotherapy and different reconstructive methods on IJVT with enrolling an adequate number of patients. 
